# Pattern and Outcome Trends in Pregnancy Related Acute Kidney Injury over 35 years: A study of 2,275 cases

**DOI:** 10.12669/pjms.42.9.16972

**Published:** 2026-09

**Authors:** Rubina Naqvi, Sehar Qureshi, Uzma Farooq, Rashid Shaikh

**Affiliations:** 1 Rubina Naqvi, Department of Nephrology, Sindh Institute of Urology and Transplantation (SIUT), Karachi. 74200. Pakistan; 2 Sehar Qureshi, Department of Nephrology, Sindh Institute of Urology and Transplantation (SIUT), Karachi. 74200. Pakistan; 3 Uzma Farooq, Department of Nephrology, Sindh Institute of Urology and Transplantation (SIUT), Karachi. 74200. Pakistan; 4 Rashid Shaikh, Department of Nephrology, Sindh Institute of Urology and Transplantation (SIUT), Karachi. 74200. Pakistan

**Keywords:** Acute Kidney Injury (AKI), Mortality predictors, Obstetrical, Pregnancy-related (PR-AKI), Renal Replacement Therapy (RRT)

## Abstract

**Objectives:**

Pregnancy related acute kidney injury (PRAKI) is a serious complication during or soon after child birth where sudden onset renal dysfunction occurs, often requiring renal replacement therapy. Its incidence and prevalence vary widely across the globe. We, at a tertiary renal care unit, while providing all renal care free of cost to patient, receive large number of patients from all over country. Present study which expands over 35 years describes largest number of PRAKI series, highlighting causes, course of illness and outcome.

**Methodology:**

This is a retrospective cohort performed at Sindh Institute of Urology and Transplantation (SIUT), Karachi, Pakistan. From January 1990 to December 2024 case records of all patients who satisfy KDIGO definition of AKI, developing it during pregnancy or soon after child birth, were reviewed and data collected and analyzed on SPSS Version26.0.

**Results:**

During this 35-year period, total 2,275 cases of PRAKI were seen at this institution, these were 24% of total AKI registered here. Mean age was 28.46 ±5.83 (range from 15-46 years). Oligo-anuria was frequent symptom found in 90.19%. main causes were peripartum excessive blood loss, intra uterine fetal death, sepsis, pre-eclampsia/ eclampsia, lower segment caesarean section and hemolytic uremic syndrome/ thrombotic Microangiopathy. The last has shown rising trends over last decade. Many women had more than one contributing factor. Renal replacement therapy required in 94%. Complete renal recovery was seen in 27%. End stage renal failure in 19%, Chronic kidney disease in 29% and follow up was missing in 14% patients. Overall mortality was 248 (10.9%), and was high in patients who were anuric, had abnormal platelet counts, and/or marked liver dysfunction and/or deranged coagulation, and/or required mechanical ventilation. Many of those who died had combination of the mentioned parameters.

**Conclusion:**

PRAKI is serious complication of pregnancy, it requires immediate address to the problem. Its markedly different (high) prevalence in developing world indicates impact of socioeconomic status and provision of basic health services in this part of world. Training of health care providers at basic health units with improved antenatal care, skilled birth attendance, timely addressing the problem and referrals to appropriate services must be considered seriously.

## INTRODUCTION

Acute kidney injury (AKI) in relation to complication occurring during or soon after pregnancy is mostly from preventable causes, but results in remarkable increase in maternal morbidity and mortality. Its incidence and prevalence varies widely among different parts of world; uncommon entity in developed world to frequent observation in some of developing countries. From developed world incidence from 1960s dropped from one in 3,000 to one in 18,000 pregnancies, while a prospective study from Nigeria recently reported incidence of 22.2%.^1^,^2^ Same country, or even same city found to report huge variation from 35%^3^ to 0.5%^4^ of total AKI, depending on various factors. A teaching hospital from Ethiopia, has reported prevalence rate of pregnancy related AKI (PRAKI) 68 per 100,000 births, with pre-eclampsia, sepsis and hemorrhage around child birth as most common causes of PRAKI.^5^ From neighboring country India changing trends from same center have been reported over a period of three decades and incidence of PRAKI declined from 15.2 to 4.68% of total AKI.^6^

While developed world reports Hemolysis, elevated liver enzymes and low Platelet (HELLP), Pre-eclampsia and hemolytic Uremic Syndrome (HUS)/ Thrombotic Microangiopathy (TMA) as common causes of PRAKI,^7^ in developing world excessive hemorrhage pre or post-partum, sepsis and intrauterine fetal death more prevailing.^3^,^8^ Several studies from the country have reported PRAKI with commonality of addressing issue of poor antenatal care, lack of health care services, pregnancy and child birth handling by untrained staff often at home, leading to high prevalence of sepsis, uncontrolled excessive hemorrhage, loss of fetus and high maternal mortality.^9^-^12^

We aim to report here a large cohort of PRAKI with determination of causes, course of illness, need for renal replacement therapy and outcome both renal and patient in this population.

## METHODOLOGY

The study is a retrospective cohort where medical records of all the patients developing AKI soon after child birth, brought to Sindh Institute of Urology and Transplantation (SIUT), between January 1990 to December 2024 were reviewed. Data was entered on Excel sheet and later shifted to SPSS for analysis. AKI was defined in retrospect according to KDIGO guidelines (abrupt decline in urine output or rise in creatinine or both).^13^

### Ethical approval:

Institutional Ethics Review Committee was approached to grant permission to see (Ref. No.: ERC 2020/A-216; dated August 10, 2020) medical records and publish results.

Complicated obstetrics with one or more than one insult was determined by clinical history. Only AKI patients with pregnancy related complications were included, while AKI from other causes or patients with chronic diseases like chronic kidney disease, long standing hypertension or diabetes mellitus patients were excluded. Demographics including age, days of insult (time of child birth to time of reporting to our institution), clinical symptoms, urine output, and laboratory investigations including hemoglobin, total leucocyte count, platelet count, peripheral film, urea, creatinine, electrolytes, calcium, albumin, lactate dehydrogenase, liver function tests, urinalysis, radiological examinations like chest roentgenogram, ultrasound kidneys (in all cases), computerized tomography/magnetic resonance imaging (in selected cases), management with fluid replacement, renal replacement therapies all recorded on Excel sheet for all patients included in study.

Renal biopsy was performed in selected cases who showed delayed renal recovery without any obvious secondary insult or had clinical parameters consistent with hemolytic uremic syndrome. Patients data was divided into four specific time blocks spanning 10 years, 10 years, five years and 10 years to analyze long term trends and pattern across multiple decades. Outcome labelled as complete recovery when serum creatinine found < 1mg/dL during follow up period, marked died when death occurred during 90 days of insult, End Stage Renal Failure (ESRF) when they remained dialysis dependent and Chronic Kidney Disease (CKD) when after 90 days of insult they had serum creatinine >1.4 mg/dL but there was no RRT requirement. Lost to follow up were the patients who did not have available serum creatinine levels up to 90 days.

### Statistical analysis:

Data was analyzed using the Statistical Package for the Social Sciences (SPSS), version 26.0 (IBM Corp., Armonk, NY, USA). Frequencies and percentages for categorical variables were computed and analyzed using the chi-square test or Fisher’s exact test.

## RESULTS

Between January 1990 to December 2024, total 2,275 PRAKI patients were seen at this institution. Demography and laboratory values from day of first registration are given in Table-I. The causes/ insults leading to PRAKI are given in Table-II, where population distributed in 10 years, 10 years, five years and 10 years, to differentiate any change in trends in causes over these years. Many patients had more than one cause contributing to injury. Only six patients from total population arrived with acute kidney injury during last trimester of pregnancy (one had HUS/TMA, two with dengue infection and delivered pre maturely bled heavily, and three with preeclampsia developed AKI before proceeding for labor induction), rest were all post-partum or post abortion.

**Table-I:** Demographic and initial laboratory in studied population.

Parameters	Mean± SD	Median	Range
Days of insult	12.09± 9.28	10	1-73
Age	28.46 ±5.83	28	15-46
Hemoglobin (11.5-15.4 G/dl)	8.21±2.30	7.95	2.3-18
WCC (4-11 x10^3^/μl)	20.37±10.35	18.5	2-105.2
Platelet (150-400 x10^3^/μl)	208.11±179.29	149	3-1546
Urea (15-39 mg/dl)	184.59±89.86	167	47-624
Creatinine (0.5-1.2 mg/dl)	12.29±3.95	11	1.45-33.15
Na (135-145 mmol/L) <135= 1003, >145=137	134.64±8.33	135	99-189
K (3.5-5.0 mmol/L) >5 = 902, <3.5 =189	4.90±1.19	4.7	1.8-9.7
HCO3 (22-30 mmol/L) ≥30 = 8, 10-22 =1786 <10=310	15.08±5.19	15	2-42
Calcium (8.5-10.2 mg/dL) >10.2 = 6 <8.5 =661	7.75±0.90	7.8	4.4-13.18
Albumin (3.5-5.5 g/DL) >5.5 = 70 <3.5 =557	2.40±0.56	2.37	0.9-5.2
LDH (140-280 U/L)	1708.33±1454.75	1374	58-19360
SGOT /AST (8-45 U/L)	185.53±490.41	47	2-6492
SGPT/ ALT (7-56 U/L)	127.06±313.18	40	2-5480

**Table-II:** Causes of PRAKI in studied population n=2,275.

Causes	1990-1999 (10 years) N=357 (22% of total AKI)	2000-2009 (10 years) N= 449 (26% of total AKI)	2010-2014 (5 years) N= 635 (35% of total AKI)	2015-2024 (10 years) N= 834 (21% of total AKI)	Total (35 years) N=2,275 (24% of Total AKI)
Septic Abortion	49 (14)	57 (13)	39 (6)	28 (3.3)	173 (7.6)
APH	113 (31.65)	133 (29.62)	180 (28.34)	277 (33.21)	703 (30.90)
Pre-Eclampsia/ Eclampsia	66 (18.48)	54 (12.02)	107 (16.85)	218 (26.13)	445 (19.56)
IUD	145 (40.61)	146 (32.51)	291 (45.82)	337 (40.40)	919 (40.39)
PPH	117 (32.77)	120 (16.03)	167 (26.29)	285 (34.17)	689 (30.28)
Sepsis	60 (16.80)	72 (16.03)	69 (10.86)	148 (17.74)	349 (15.34)
HUS / TMA	4 (1)	18 (4)	12 (2)	137 (16.4)	169 (7.42)
HELLP	22(6.1)	32 (7.1)	48 (7.5)	68 (8.1)	170 (7.47)

%given in parenthesis. Count exceeds to 100 as there was frequent overlap of causes (one woman had more than one contributing factor at same time).

On similar accounts with time distribution outcome of this population in complete renal recovery, death, ESRF, partial recovery (CKD Stages 2-4) and lost to follow up before 90 days of insult. Lost to follow up was highest (29%) during last ten years, though many of these were showing improvement at time of discharge in terms of urine output or leveling off serum creatinine but their final report of serum creatinine up to 90 days is not available Table-III.

**Table-III:** Outcome during different time periods (% in parenthesis).

Time Duration	Complete recovery	CKD	Lost to Follow up	ESRF	Death during acute illness
1990-1999 (10 yr) n = 357	146 (41)	66 (19)	14(4)	80 (22)	51 (14)
2000-2009 (10yr) n = 449	130 (29)	132 (29)	40 (9)	104 (23)	43 (10)
2010-2014 (5yr) n = 635	185 (29)	185(29)	77 (12)	106 (17)	82 (13)
2015-2024 (10yr) n= 834	165 (20)	205 (25)	246 (29)	146 (17)	72 (9)
Total	626 (27.51)	588 (25.84)	377 (16.57)	436 (19.16)	248 (10.9)

Most of the patients (85.9%) reached to this institution 1-14 days (up to two weeks) after child birth, and only 3% had duration of insult for more than four weeks. Decline in urine output was most common presenting symptom, with 90.1% presenting with oligo-anuria. Half of patients (50.2%) had thrombocytopenia, and a small number (13.8%) had thrombocytosis. Abnormal INR was found in 16.9%. Severe acidosis that is, venous bicarb <10 was present in 13.6% of patients, while 78.5% had mild or moderate acidosis (venous bicarb between 10-22). Abnormal liver functions with elevated bilirubin levels were seen in 32.2% and 36.8% had elevated liver enzymes with or without affecting bilirubin levels. Mechanical ventilator support was required in 7.4% patients.

Patients who presented late (>4 weeks) to the hospital had a higher incidence of ESRF (8%) compared to patients who recovered from their kidney injury (1.4%). The vast majority of patients with poor outcomes (death 98.2%, ESRF 98.5%, and CKD 97.6%) found to have oligo-anuria (P<0.005). Thrombocytopenia was significantly more common among patients who died (64.9%) versus those who survived (48.6%) or developed ESRF (41.3%) (P<0.005), indicating an association with increased mortality. Thrombocytosis was also significantly associated with increased mortality. A strong association existed between deranged INR levels (P<0.005), found in over half of deaths (53.1%) as opposed to approximately 17-19% in other groups.

Mortality was strongly associated with markers of liver damage, abnormal bilirubin (59.3% of deaths vs 22.7% – 33.9% of others) and elevated liver enzymes (58.3% of deaths vs 30.7% of recovered patients) Table-IV. The requirement of mechanical ventilation exhibited the strongest association (P<0.005); 52% of deaths were on ventilator support compared to minimal proportions in the recovered (3.7%), ESRF (0.9%), and CKD groups (1.7%). In total, the mortality of patients with a duration of insult of 1-2 weeks was 12.8%, duration of insult of 2.1-4 weeks 12.6%, and duration of insult of greater than four weeks 7.6%; however, differences did not reach statistical significance (P=0.42), demonstrating that the duration of insult itself was not significantly associated with mortality. But when compared with those who recovered 1.4% of them reached at >4 weeks of insult vs 8% of ESRF arrived late. Acidosis status associated with higher mortality, especially in severely acidotic patients 16.2% who recovered vs 20.2% of those who died, but the relationship was not statistically significant Table-IV.

**Table-IV:** Comparison of variables among recovered and died population.

Variables	Total	Died	Recovered	P-Value
Duration of Insult	874	n=248	n=626	
1 to 2 weeks	777	215(86.7%)	562(98.8%)	0.420
2.1 to 4 weeks	83	28(11.3%)	55(8.8%)	
>4 weeks	14	5(2%)	9(1.4%)	
Oligo anuria	817	n=218	n=599	
Yes	762	214(98.2%)	548(91.5%)	0.001
No	55	4(1.8%)	51(8.5%)	
Thrombocytopenia	874	n=248	n=626	
Yes	465	161(64.9%)	304(48.6%)	<0.005
No	409	87(35.1%)	322(51.4%)	
Thrombocytosis	874	n=248	n=626	
Yes	118	21(8.5%)	97(15.5%)	0.006
No	756	227(91.5%)	529(84.5%)	
Derange INR	703	n=211	n=492	
Yes	205	112(53.1%)	93(18.9%)	<0.005
No	498	99(46.9%)	399(81.1%)	
Acidosis status	765	n=223	n=542	
Severe Acidosis	134	45(20.2%)	89(16.4%)	0.214
Mild to moderate Acidosis	631	178(79.8%)	453(83.6%)	
Abnormal Bili	775	n=221	n=554	
Yes	319	131(59.3%)	188(33.9%)	<0.005
No	456	90(40.7%)	366(66.1%)	
Liver Enzymes	784	n=223	n=561	
Normal	482	93(41.7%)	389(69.3%)	<0.005
High liver enzymes	302	130(58.3%)	172(30.7%)	
Required Ventilator	874	n=152	n=722	
Yes	152	129(52%)	23(3.7%)	<0.005
No	722	119(48%)	603(96.3%)	

The relationship between clinical and laboratory parameters and the development of ESRF is shown in Table-V. The length of time the patient has been insulted had a significant relationship with ESRF (P<0.005) and a strongly increasing trend in ESRF as duration increased: 1–2 weeks (19.5%), 2.1–4 weeks (32.9%), >4 weeks (53%), which supports the association between delayed presentation and frequency of progression to ESRF. Furthermore, oligo-anuria, thrombocytopenia, deranged INR, abnormal liver functions and respiratory distress were significant predictors for patients developing ESRF if survived.

**Table-V:** Association of Clinical Characteristics and Laboratory Parameters with ESRF.

Variables	Total	ESRF (n= 436)		P-Value
		Count	Percent	
***Duration of Insult (n=1966)***				
1 to 2 weeks	1678	328	19.5%	P<0.005
2.1 to 4 weeks	222	73	32.9%	
>4 weeks	66	35	53%	
***Oligo anuria (n=1857)***				
Yes	1781	402	22.6%	0.002
No	76	6	7.9%	
***Thrombocytopenia (n=1966)***				
Yes	982	180	18.3%	P<0.005
No	984	256	26%	
***Thrombocytosis (n=1966)***				
Yes	285	71	24.9%	0.229
No	1681	365	21.7%	
***Derange INR (n=1496)***				
Yes	344	52	15.1%	0.008
No	1152	250	21.7%	
***Acidosis status (n=1699)***				
Severe Acidosis	277	62	22.4%	0.810
Mild to moderate Acidosis	1422	309	21.4%	
***Abnormal Bili (n=1701)***				
Yes	563	80	14.2%	0.0005
No	1138	272	23.9%	
***Liver Enzymes (1723)***				
Normal	1085	240	22.1%	0.073
High liver enzymes	638	118	18.5%	
***Required Ventilator (n=1966)***				
Yes	167	4	2.4%	0.0005
No	1799	432	24%	

Associated co morbid in these women were; malarial parasite seen in 33 women, it was Falciparum in 14, vivax in 18, both falciparum and vivax in one, species not specified in seven. Acute gastroenteritis a day before child birth or soon after was reported in five women.

Renal biopsy was carried out in 237 women, who either showed delayed renal recovery or had features of HUS, pure acute tubular necrosis was found in 24, extensive cortical infarction in 11, focal cortical infarction in 32, features of HUS/ thrombotic Microangiopathy (TMA) found in 164 (56 of these had associated extensive and 77 focal cortical infarction along with TMA), tubulo-interstitial and glomerular changes seen in three each. Fig.1

**Fig.1:**
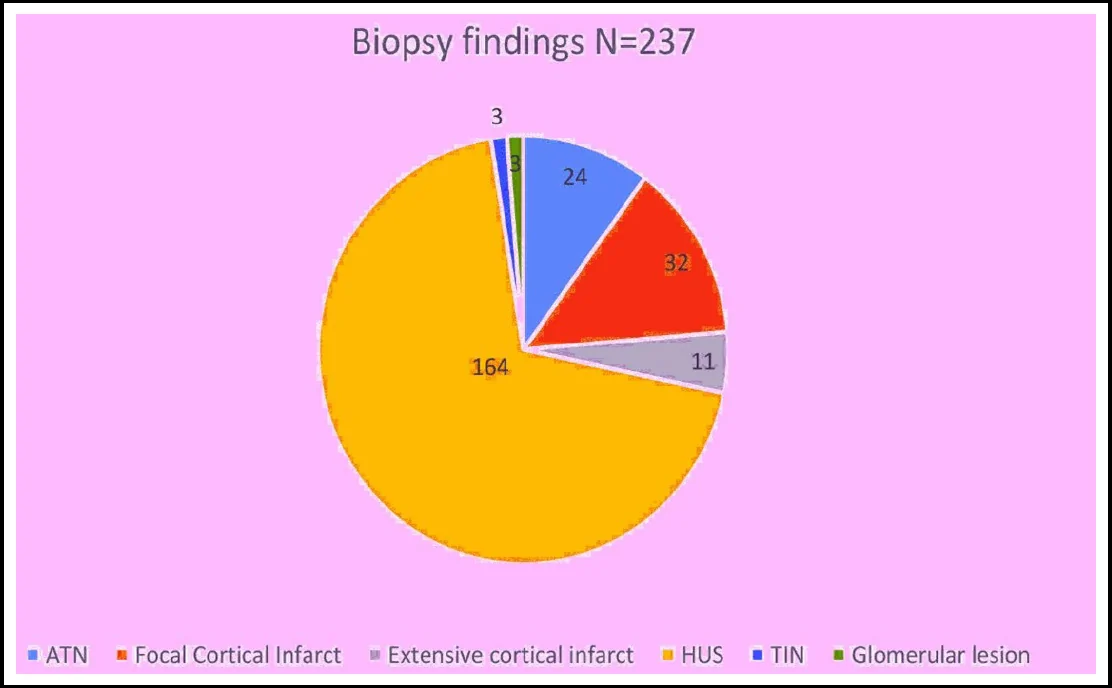
Biopsy Findings. ATN=24, Extensive cortical infarction=11, Focal cortical infarction =32, HUS total=164 (Extensive cortical infarction with HUS=56, Focal cortical infarction with HUS= 77, isolated HUS=24, HUS+ATN=7), TIN=3, Glom lesion=3.

## DISCUSSION

Over past 3.5 decades PRAKI has evolved at our institution in term of increase in number of overall AKI patients coming to this institution. PRAKI over initial ten years total of 357 cases which were 22% of total AKI, to 834 PRAKI during last reported decade which is 21% of total AKI. So patients’ turnover has increased but PRAKI contributing to total AKI not much changed. Highest PRAKI was observed during 2010-2014 when it was 35% of total AKI (Table-II).

Changing trends in a study joint publication from North and Western India reports PRAKI requiring RRT has fallen from 98 to 66 % over a span of 33 years.^6^ Another study from South India expressing changing pattern in title have actually reported number of PRAKI seen and managed over ten years’ period and not have compared any change in pattern of causes. This particular study has compared many other studies published from India.^14^

Our present study has shown change in pattern in regard of septic abortions which were 14% during initial ten years and dropped to 3.3% over last ten years. This particular change has no logical reasoning as in Pakistan it is still illegal to go for induced abortion other than certain medical reasons threatening life of women. Thus, induced abortions for unwanted pregnancies still go for some homemade remedies or unskilled personnel performing at back door with no proper sterile environment. Another change observed in our present study is marked rise in HUS/TMA in this population which was 1% during initial ten years and 16.4% during last ten years. Although TMA in relation to pregnancy and post-partum period is widely addressed in literature with possibility of multiple mechanisms involvement.^15^

However, the reason for marked rise in number of these patients in current study is not clear, the shift could be due to more frequent use of diagnostic kidney biopsies, greater clinical awareness of pregnancy related HUS, and pregnancies at an advanced maternal age and the complex physiological triggers of the postpartum period, that cause uncontrolled immune system activation in genetically predisposed women can trigger complement-mediated kidney injury. Among 237 renal biopsies done in women in present study, 164 (69.19%) found to have TMA either with focal or extensive cortical infarction, as shown in Fig.1

Apart from regional other low income countries also report PRAKI, A teaching hospital from Ethiopia, has reported prevalence rate of pregnancy related AKI (PRAKI) 68 per 100,000 births, with pre-eclampsia, sepsis and hemorrhage around child birth as most common causes of PRAKI.^5^ At this teaching hospital OBGYN unit they were looking at prevalence of AKI in women who were registered during antenatal and coming to a teaching hospital setup where chances of skilled and trained health care workers’ availability were high. There must be reports dealing with unregistered pregnant women at private setup and complications occurring never reported in literature.

The incidence, characteristics, and outcomes of AKI in general across the African continent are under-reported. A published review which was conducted to highlight the clinical profile and outcomes of PR-AKI in Africa, search results across the 54 African countries could only found 14 studies accessible which met the eligibility criteria.^16^

From neighboring country India Yadla et al. over ten years reported 500 PRAKI, but their categories did not mention peri partum hemorrhage. They have rather categorized as preeclampsia in 21, sepsis in 26 and combination of both in 34% women.^8^ Another study from India reporting 165 PRAKI have seen ante or post-partum hemorrhage in only two cases,^17^ these reports have wide variation from our series where 61% women had either ante or post-natal (or combination of both) hemorrhage and multiple blood and blood products transfusions.

Complete renal recovery in our PRAKI series is lower than compared to our other AKI causes^18^,^19^ this may be related to combination of insults in same women ischemic acute tubular necrosis or cortical necrosis after excessive blood loss along with surgical trauma of lower segment caesarean section along with sepsis, which then superadded by nephrotoxins; antimicrobials and analgesic and frequent use of proton pump inhibitors which are proven nephrotoxins.^20^ Also those who require hemodialysis, there is combination of hemodynamic changes, inflammatory stimuli, oxidative stress, cascade of cytokine upsurge and vasoconstriction adding to ischemic insult and halting recovery.^21^

In our studied population 94% patients required RRT, this can be well explained for the reason that ours is tertiary care unit and providing all renal care facility free of cost, thus patients from all over country referred to this institution and they often reach with advanced uremia. For need of RRT others have reported 30%^17^ to 75%.^14^ Same argument of tertiary care unit and thus patients reaching here are mostly in advanced uremia and have combination of problem, can also be true for mortality in 11% and development of ESRF in another 19%. Unfortunately, complete renal recovery was low (27.5%) in our population, which is different from other reports of 33 to 81%, in different stages of AKI at time of presentation.^17^

Another important observation regarding change in our population trends was sizeable population during over last ten years did not show up for suggested follow-up (Table-III), though about half of them were showing improvement in terms of urine output and serum creatinine but uncertainty remains about complete or partial recovery in these patients. This phenomenon could be result of expansion of services at this particular institution, to 4 to 5 fold with rapid patient turnover, this may cause patient dissatisfaction and reluctance to come back. Furthermore, long term retrospective cohorts tend to show decline in follow up trends.

Overall the study population is young reproductive age women and lack of proper ante-natal care, child birth at home or small setup with untrained/ unskilled worker, left them with serious complication of AKI which remain irreversible in many or even ended in death in some. Delay in referral to proper health care set-up or in selected cases to renal care facility, all lead to sizeable number of mortality with a cause which in most cases is preventable or reversible. All we are bringing here is just “tip of iceberg” and number of peri-partum mortality must be much higher with unreported cases from rural Pakistan. Improving ante natal care is most cost effective way of reducing PRAKI.

At level of a standardized state run hospital a multidisciplinary approach, nephrologists and obstetrician working together can recognize AKI at an early stage,^22^ even before any change in serum creatinine or urine output in risk population, can also reduce the number of established PRAKI and further its undesired course.

### Limitations:

being a retrospective study over a long duration of 35 years, there are always possibilities of missing data and follow up from available records. Those who had provided contact numbers at time of initial registration approached and sometimes called to check their general health and laboratory parameters. But as we cater patients from far flung and remote rural areas many could not contacted.

## CONCLUSION

PRAKI is a major public health issue in Pakistan. It is strongly recommended to design and implement “PRAKI Prevention Bundles”. This includes good ante natal at basic health units in rural areas, with regular BP monitoring, early Ante and post-partum hemorrhage detection, recognition of problem and knowledge about when and where to refer the patient, antibiotic stewardship and avoidance of nephrotoxins are the core concerns to address.

### Authors’ Contribution:

**RN:** Data collection, Literature search, study design and manuscript writing, manuscript revision, validation and is responsible for the integrity of the study.

**UF, RS and SQ:** Patient management.

**SQ:** Data analysis.

All authors have read and approved the final version of the manuscript.
